# Sepsis From Multisystem Infection With Multidrug-Resistant Raoultella ornithinolytica

**DOI:** 10.7759/cureus.20975

**Published:** 2022-01-05

**Authors:** Abayomi Tayo, Kwaku Nyame

**Affiliations:** 1 Emergency Medicine, Komfo Anokye Teaching Hospital, Kumasi, GHA

**Keywords:** ghana, infectious, raoultella ornithinolytica, multidrug resistance, antibiotic resistance, sepsis

## Abstract

*Raoultella (R.) ornithinolytica* is a gram-negative, encapsulated aerobe or facultative anaerobe belonging to the family *Enterobacteriaceae*. It is distinguished from other members in the family with a negative indole test, growth at 10^o^C, production of histamine, a negative D-melezitose test, and metabolism of ornithine. *R. ornithinolytica* is a versatile organism found in different habitats, including soil, water, and plants, as well as colonizing the human gastrointestinal tract and throat. It was considered to cause opportunistic infection in humans but is increasingly being implicated in infections in immunocompetent individuals. *Raoultella ornithinolytica* causes infection in different clinical settings. Sepsis from *Raoultella ornithinolytica* is increasing among humans, and it is considered an emerging infectious agent in both immunocompromised and immunocompetent people. There is an increasing level of antibiotic resistance among isolates with reports of multidrug resistance.

We report the case of a 95-year-old man with multidrug-resistant *R. ornithinolytica* multisystem infection and review the literature.

## Introduction

*Raoultella (R.) ornithinolytica* is a gram-negative aerobic or facultative anaerobic, encapsulated bacilli belonging to the family of *Enterobacteriaceae*. They are found in natural habitats like plants, water, and soil. They colonize insects, termites, ticks, and some fish. They have also been found to colonize the gastrointestinal tract and throat of humans. *R. ornithinolytica* has been implicated in histamine poisoning [1-5].

*R. ornithinolytica* infections were considered opportunistic infections in humans with comorbidities, especially immunosuppression. They were not known to infect immunocompetent humans. However, infections have been found to have occurred in immunocompetent and immunocompromised individuals as well. It has also been found to cause disease in community-acquired infections as well as nosocomial infections in patients who had undergone invasive procedures. Immunocompromised individuals include patients with diabetes mellitus, oncology patients, and patients with solid organ transplants on immunosuppressive therapy and the extremes of ages [1,3,6-10].

*R. ornithinolytica* has been described to cause gastrointestinal and biliary disease; ear, nose, and throat infections; joint infections; urological infections; central nervous system infections; pneumonia; as well as infections during medical instrumentation. The antibiotic sensitivity pattern and disease outcomes are very varied. *Raoultella spp*. is intrinsically resistant to penicillin due to the production of broad-spectrum beta-lactamase. There are several acquired genes described in them, leading to increasing antibiotic resistance and multidrug resistance. There are few reported cases of multidrug resistance *R. ornithinolytica* [1,2,5,11-13].

We report the case of a 95-year-old man with a multidrug-resistant *R. ornithinolytica* multisystem infection. 

## Case presentation

A 95-year-old male, with no previously known chronic medical condition but a smoker for 20 years, presented to the emergency department (ED) with a three-day history of fever and a day’s history of altered level of consciousness.

The patient was well until three days before presentation to the ED when he started experiencing high-grade fever and persistent headache. He was, however, found the next day to be generally weak and unable to get up from bed but was responsive. The patient was given over-the-counter medications for the fever, but the patient deteriorated and developed an altered level of consciousness on the day of presentation and was brought to the ED. This was the first episode of such and there were no reported complaints of chest pain, difficulty in breathing, palpitations, vomiting or diarrhea, abdominal pain, or neck pain.

On examination at the ED, the patient was semi-conscious with a Glasgow Coma Score of 8/15 and warm to touch with a temperature of 39.1^o^C, respiratory rate of 26 cycles per minute with oxygen saturation of 97% on room air. The pulse rate was 115 beats per minute with a blood pressure of 146/96 mmHg. There was no neck stiffness and a negative Brudzinski sign. Chest examination revealed reduced air entry bilaterally with crackles at the posterior lung bases. There were no significant findings on abdominal examination. The patient was immediately started on empirical antibiotics (ceftriaxone and azithromycin).

Rapid diagnostic testing for malaria (both *Plasmodium falciparum* and mixed *Plasmodium spp.*) was negative. Also, rapid diagnostic tests for Hepatitis B and C were negative. The complete blood count indicated an elevated white cell count with neutrophilia and lymphopenia. A repeat of the complete blood count on Day 4 of admission indicated a higher level of neutrophilia with a much-reduced lymphocyte count. Antibiotics adjusted accordingly to intravenous (IV) amikacin 500 mg daily after the culture and sensitivity results. The patient passed away two days after the start of the amikacin, on the seventh day of admission. See Tables 1-2.

**Table 1:** Blood and Urine Culture Results and the Complete Blood Count Results MCV: mean corpuscular volume; MCH: mean corpuscular hemoglobin

Cultured Sample Source				Complete Blood Count			
					Day One	Day Four	
Antibiotic	Right Arm	Left Arm	Urine	Parameter	Units	Units	Reference Range
Amikacin	Sensitive	Sensitive	Sensitive	Hemoglobin	12.9g/dl	13.5g/dl	12.0– 18.0g/dl
Imipenem	Resistant	Resistant	Resistant	RBC Count	4.79x10^6/uL	5.07x10^6/uL	3.00-5.00x10^6/uL
Cefepime	Resistant	Resistant	Resistant	Hematocrit	40.70%	42.80%	26.0 - 50.0%
Tetracycline	Resistant	Resistant	Resistant	MCV	85 fL	84.4fL	80.0 – 100.0fL
Aztreonam	Resistant	Resistant	Resistant	MCH	26.9 pg	26.6 pg	26.0 – 38.0 pg
Chloramphenicol	Resistant	Resistant	Resistant	White Cell Count	12.73x10^3/uL	12.54x10*3/uL	4.0–10.0x10*3/uL
Trimethoprim/sulfamethoxazole	Resistant	Resistant	Resistant	Neutrophil	10.28x10^3/uL (80.7%)	11.04x10^3/uL (85.9%)	1.50-7.00x10^3/uL (37 – 72%)
Gentamycin	Resistant	Resistant	Resistant	Lymphocytes	1.01x10^3/uL (7.9%)	4.9x10^3/uL (4.9%)	1.00-3.70x10^3/uL (20- 50%)
Amoxycillin/Clavulanic Acid	Resistant	Resistant	Indeterminate	Monocyte	1.42x10^3/uL (11.2%)	1.15x10^3/uL (9%)	0.00-0.70x10^3/uL (0 - 14%)
Ceftazidime	Resistant	Resistant	Indeterminate	Eosinophils	0.01x10^3/uL (0.1%)	0.01x10^3/uL (0.1%)	0.00-0.40x10^3/uL (0.0-6.0%)
Ciprofloxacin	Resistant	Resistant	Resistant	Basophils	0.01x10^3/uL (0.1%)	0.01x10^3/uL (0.1%)	0.0-0.10x10^3/uL (0.0 - 1.0%)
Cefuroxime	Resistant	Resistant	Resistant	Platelet	128x10*3/uL	133x10*3/uL	140-440 x10*3/uL

**Table 2:** Urine Biochemistry and Microscopy

Urine Biochemistry	Results	Urine Microscopy	Results (Reference range)
Urobilinogen	2+ (66 umol/L)	WBCs	2224.1/uL (0 - 23/uL)
Blood	2+ (25 cell/uL)	RBCs	13.3/uL (0 - 23/uL)
Bilirubin	1+ (8.6 umol/L)	Hyaline Cast	0.97/uL (0 - 1/uL)
Protein	2+ (1.0 g/L)	Bacteria	84761.5/uL (0 - 1200/uL)
pH	5.5	Yeast Cells	61.9/uL (0 - 1/uL)
Nitrite	Negative	Crystals	11.1/uL (0 - 10/uL)
Leucocytes	3+ (500 cells/uL)		
Glucose	Negative		
Ketones	Negative		

## Discussion

We present a 95-year-old man with no known previous medical condition but sepsis from a multisystem infection with multidrug-resistant *Raoultella ornithinolytica*.

*Raoultella ornithinolytica* is a gram-negative nonmotile aerobic but facultative anaerobic encapsulated bacilli belonging to the *Enterobacteriaceae* family of bacteria. They form large mucoid colonies on McConkey agar due to the synthesis of the polysaccharide capsule. Its distinguishing features include a negative indole test, growth at 10^o^C, production of histamine, negative D-melezitose test, and metabolism of ornithine. They are found in common places like plant sab, water, and soil. They are also found to colonize some insects like bees, termites, and ticks. *R. ornithinolytica* are versatile bacteria [1-4].

*R. ornithinolytica* has been implicated in scombroid syndrome due to the production of histamine in certain species and is related to the consumption of tuna, bonito, sardines, and mackerels that are stored poorly [1,3-4]. They have been found to also colonize humans in the throat and have been identified in human saliva and the gastrointestinal tract. They have also been isolated from the hospital environment [1-2,4]. There is a reported case of *Raoultella ornithinolytica* isolated from chicken products in the market [5].

*R. ornithinolytica* was initially considered not to infect humans and considered to cause infection in immunocompromised individuals with diabetes mellitus, patients with solid organ transplants on immunosuppressive therapy, and oncology patients. They have also been described in the extremes of age [6-8]. *R. ornithinolytica* is now increasingly being linked with infection in immunocompetent people without an underlying disease and is considered an emerging human pathogen [1]. Our patient was aged but without any known medical condition and did not show signs and symptoms of underlying disease. It is known to cause community-acquired infections, as well as nosocomial infections in patients who have undergone invasive procedures [8]. *R. ornithinolytica* has been implicated in localized infections of and some reported cases of sepsis and septic shock [3,9]. They have been implicated in community-acquired cases of pneumonia, hospital-acquired cases of pneumonia, and ventilator-associated cases of pneumonia, cystitis, cholecystitis, pyelonephritis, surgical site infections, and peritonitis [8]. They are also reported to be implicated in ENT infections, CNS infections, urological infections, and joint infections [8,10]. There are reported cases of bacteremia and sepsis from *R. ornithinolytica *[1,8,11]. Our patient was an elderly man with sepsis evidenced by a respiratory rate of 26 cycles per minute, fever of 39.1^o^C, and Glasgow Coma Score of 8/15 with culture positive for *Raoultella ornithinolytica* bacteria from the blood and the urine as well as bacteriuria as shown in Tables 1-2. There was clinical evidence of community-acquired bilateral lower lobe pneumonia.

*R. ornithinolytica *are intrinsically resistant to penicillin due to the production of broad-spectrum beta-lactamase even though they are described to be generally sensitive to antibiotics. *R. ornithinolytica* infections typically have been treated with third- to fourth-generation cephalosporins or fluoroquinolones alone or with aminoglycosides [1-2,4]. There are also reported cases of carbapenemase-producing *R. ornithinolytica* [12]. There are reported cases of acquired genes leading to increasing antibiotic resistance in *R. ornithinolytica* [1]. There is increased reporting of multidrug-resistant *R. ornithinolytica* [2,6]. This makes infection with and the treatment of *Raoultella ornithinolytica *troubling trend with increased morbidity and mortality. The isolate from our patient showed multidrug resistance as shown in Table 1 with resistance to many of the commonly used and readily available antibiotics making treatment difficult. This might have contributed to the mortality of the patient. There is increasing bacterial resistance to commonly available antibiotics by the development of resistant genes and the spread of these genes among species [13]. This is presenting a challenging clinical environment for the management of bacterial infections. Seng et al. reported a high mortality rate of about 20% (34%-44% in bacteremia) from *R. ornithinolytica* infection [8]. Proper identification in hospitals and healthcare settings is important for the improvement in case management.

## Conclusions

This report adds to the disturbing emergence of multidrug-resistant *Raoultella ornithinolytica* causing infection and sepsis in immunocompetent individuals. *Raoultella ornithinolytica* infection in immunocompetent individuals is increasing and causes multisystem infection with sepsis. The production of broad-spectrum beta-lactamase and acquired antibiotic resistance from gene transfer is resulting in increased drug resistance in the organism. There is the need to culture all bodily fluids promptly to direct antibiotic therapy in sepsis. There should be increased advocacy in antibiotic use stewardship to help reduce the emergence of bacterial resistance to the currently available antibiotics and to reduce the increasing trend of multidrug-resistant bacteria causing diseases.
